# Joint preservation surgery in grade 2 and 3 giant cell tumors of bone around the knee

**DOI:** 10.1051/sicotj/2021049

**Published:** 2021-09-14

**Authors:** Saurabh Singh, Alok Rai, R Dinesh Iyer, Rishabh Surana, Divyansh Sharma

**Affiliations:** 1 Professor, Department of Orthopaedics, Institute of Medical Sciences, Banaras Hindu University 221005 Varanasi Uttar Pradesh India; 2 Department of Orthopaedics, All India Institute of Medical Sciences 110029 New Delhi India; 3 Senior Resident, Department of Trauma and Emergency (Orthopaedics), All India Institute of Medical Sciences Raipur 492099 Chhattisgarh India; 4 Senior Resident, Department of Orthopaedics, Institute of Medical Sciences, Banaras Hindu University 221005 Varanasi Uttar Pradesh India; 5 Senior Resident, Department of Orthopaedics, All India Institute of Medical Sciences 249203 Rishikesh Uttarakhand India

**Keywords:** Giant cell tumor, Curettage, Joint preservation, Bone grafting, GCT

## Abstract

*Objective*: To evaluate the clinical and functional outcomes of joint preservation surgery in high-grade giant cell tumors (GCT) around the knee joint. *Methods*: A retrospective review of 25 patients of high-grade GCT (Campanacci grade 2 and 3) involving proximal tibia or distal femur managed by extended curettage, bone grafting, and stabilization with knee spanning external fixator between 2016 and 2018 was done. The radiographic outcomes, functional outcomes (Musculoskeletal Tumor Society [MSTS] score for lower limb), and complications including donor site morbidity were evaluated. *Results*: The mean age of the patient population was 24.04 years with an average follow-up period of 30.24 months. Fourteen patients had involvement of distal femur, and 11 involved proximal tibia. There were 16 cases of grade 2 lesions and 9 cases of grade 3 lesions. Twenty-four out of the 25 patients had radiological consolidation of graft, while one patient had graft subsidence. Twenty-two out of 25 patients had full extension and knee flexion more than 100 degrees. The mean MSTS score was 25.2. Three patients had an MSTS score under 20. All three patients had an extension lag with a restricted range of motion. *Conclusion*: Joint preservation surgery, when done in line with the basic principles of tumor surgery, gives good radiographic and functional outcomes even in grade 2 and 3 giant cell tumors of bone around the knee and should be attempted before replacement surgeries.

## Introduction

Giant cell tumor (GCT) of bone is a common affliction constituting about 5% of all primary bone tumors involving the meta-epiphyseal region and affecting patients mostly in the second and third decade of their lives. Though a benign tumor, it acts as a locally aggressive disease with the potential to metastasize, making it one of the most devastating diseases of young adults. The most common location is around the knee joint, i.e., distal femur and proximal tibia [1, 2]. Replacement with custom-made tumor prosthesis after resection has significantly increased in the recent decades because of less chance of recurrence, early mobilization, and restoration of joint function. Though it appears to give good results in the short term [3–5], the long-term follow-up results and its survivorship data paint a different picture, especially in young patients who have more than 40 years of life expectancy after the procedure [6–8].

Keeping in mind the principles of tumor surgeries and intralesional curettage, joint preservation surgeries coupled with local ablative methods against tumor cells like a high-speed burr, phenol, liquid nitrogen, and cauterization have given good results even in high-grade lesions. Though it does not allow immediate mobilization and early rehabilitation like replacement surgeries and requires frequent follow-up visits, which may be exhausting for that patient and their families, it does a great job of preserving the native joint and has better outcomes in the long term.

Most of the existing literature on the subject has pointed out the higher recurrence rates in joint preservation surgeries [9–12]. However, there is a lack of literature documenting the functional outcomes in cases where recurrence did not occur and the benefits of retaining a native joint compared to a prosthetic joint.

This study aimed to retrospectively review the radiographic and functional outcome of joint preservation surgeries in cases of Campanacci grade 2 and 3 GCT of bone around the knee joint.

## Materials and methods

With institutional review board approval, a retrospective study was performed at an academic medical center attached to a tertiary care hospital to evaluate the results of joint preservation surgery (curettage, autologous bone graft, and stabilization with knee spanning external fixator) in cases of Campanacci grade 2 and 3 giant cell tumor of bone around the knee joint.

### Patient selection

Records of 36 patients diagnosed with GCT of bone around the knee operated at our institution between 2016 and 2018 were screened for the study. The inclusion criteria were patients over 18 years of age with biopsy-proven GCT around the knee (involving distal femur or proximal tibia) and radiographic staging of Campanacci grade 2 and 3 who were managed by joint preservation surgery during the study duration. We excluded the patients with pulmonary or any other distant metastasis, GCTs with a fracture at the time of presentation, GCT involving patella or tendons around the knee joint, recurrent GCTs, and revision surgeries. After screening the records using the above-mentioned criteria, a total of 25 patients were included in our study. Out of these 25 patients, there were 14 females and 11 males with a mean age of 24 years. The distal femur was involved in 14 patients and proximal tibia in 11 patients.

The 11 patients excluded had two patients of pulmonary metastasis, three patients of pathological fracture of the distal femur at the time of presentation, one patient of GCT involving patella, and five patients of GCT recurrence, of which two patients underwent revision surgeries.

### Surgical procedure

**Figure 1:**
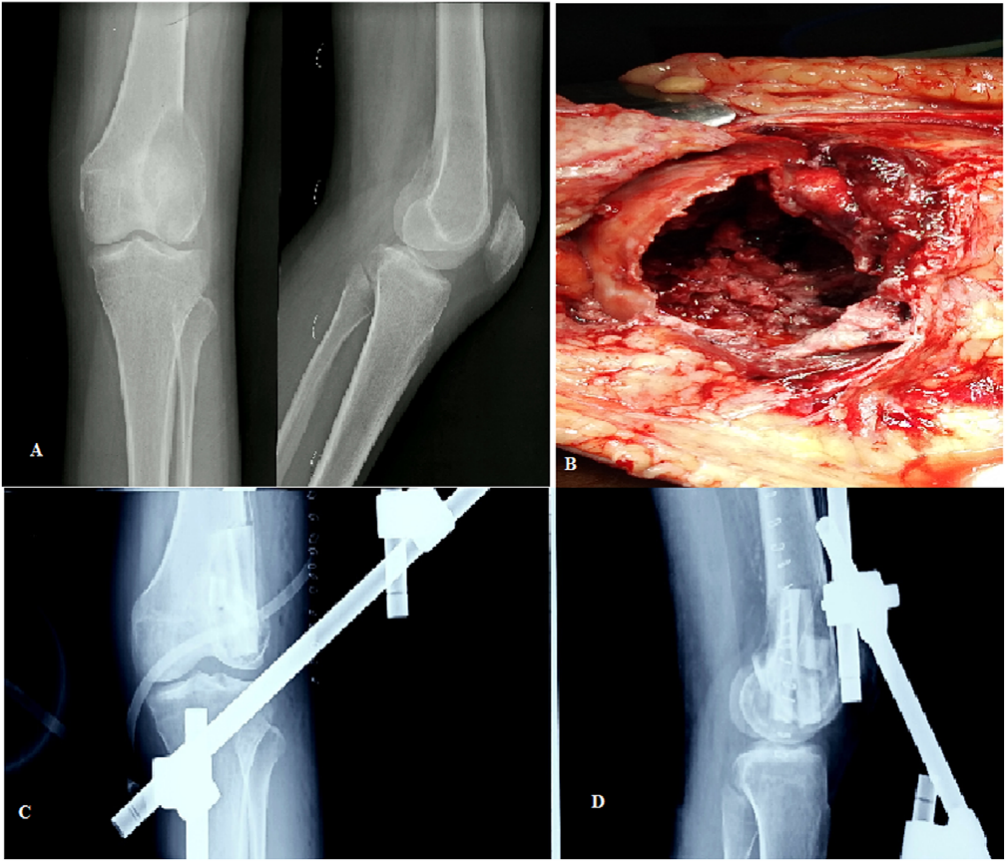
(A) Pre-operative radiographs showing lytic lesion destroying the lateral femoral condyle with a thin cortical rim; (B) Intra-operative picture of the defect after extended curettage; (C) and (D) Postoperative AP and lateral radiographs.

**Figure 2:**
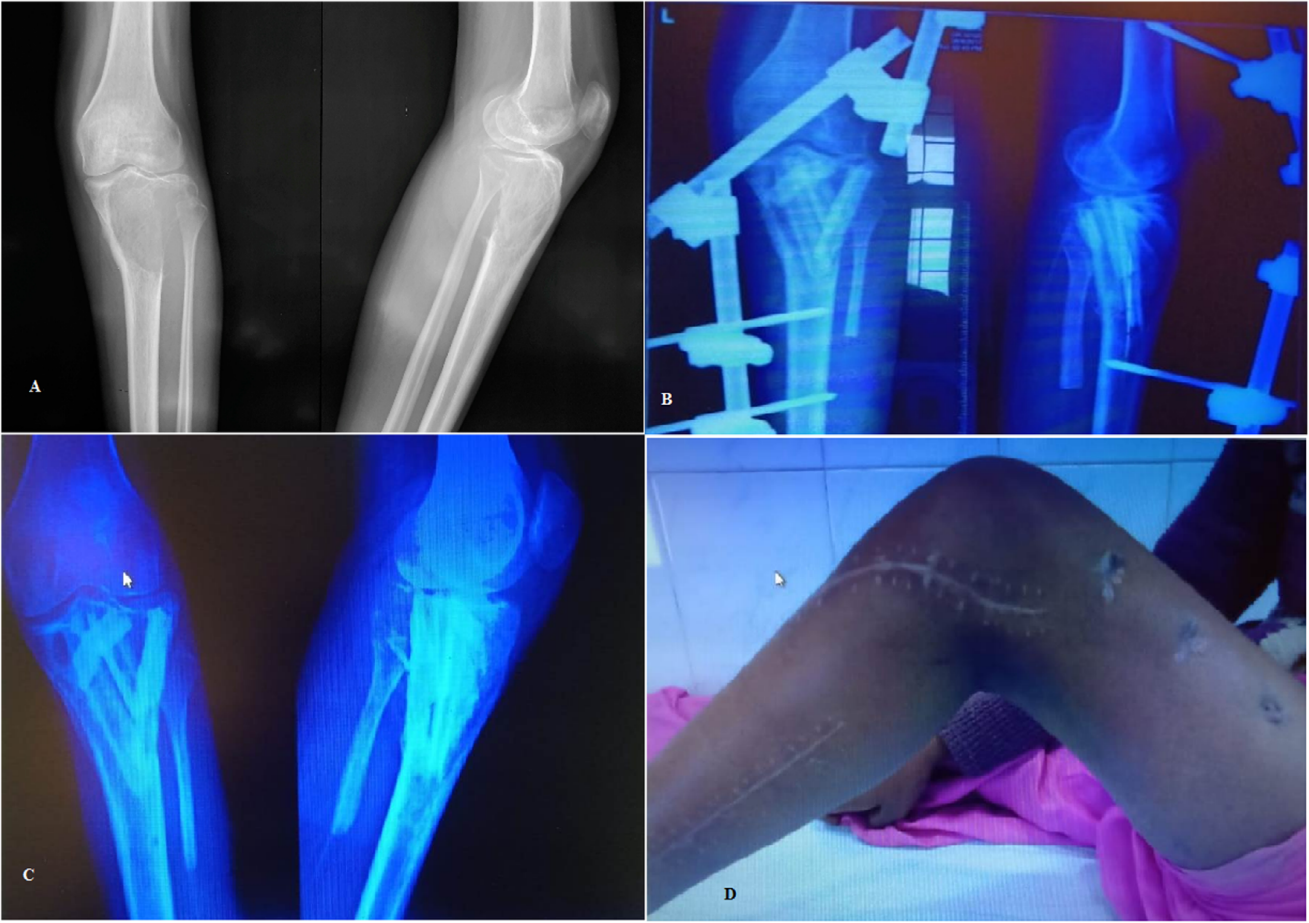
(A) Pre-operative radiographs of a Campanacci grade 3 GCT involving the proximal tibia; (B) Postoperative radiographs; (C) Follow up radiograph showing good graft uptake and no signs of tumor recurrence or penetration into joint; (D) Knee range of motion beyond 90 degrees at follow up.

All patients were operated on under spinal anesthesia with a pneumatic tourniquet placed on the thigh. For cases of distal femur involvement lateral or medial approach was used depending on the condyle involved. Similarly, for cases involving proximal tibia, anterolateral or anteromedial approach was taken as per condyle involvement. Intralesional curettage was done through a large cortical window (which was already present in most cases due to breach in the cortex). Curettage was augmented using electrocautery, high-speed burr, and liquid phenol in all our cases. We visually inspected the cavity and used a dental mirror to view inaccessible regions to look for any tumor bed that the naked eye may have left out. After curettage, cancellous graft harvested from the iliac crest was morselized and put adjacent to the articular cartilage. Fibular cortical strut graft (from the ipsilateral leg or bilateral depending on the length and number of struts required) was cut to the appropriate length to span the long axis of the defect and act as structural support. However, the whole volume of the defect was not filled in any of the cases. Stabilization was achieved using a knee spanning external fixator in all patients (Figures 1 and 2).

### Postoperative protocol and follow-up

Postoperative protocol was uniform for all patients. Patients were followed up at two weeks after surgery when sutures were removed and at 12 weeks when the external fixator was removed after radiograph, and passive knee mobilization began under supervision with the help of continuous passive motion machines. Partial weight-bearing was also begun with walker support. Complete weight-bearing was allowed after confirmation of radiographic consolidation, especially along the posterior cortex, usually around 22–26 weeks (mean 25.2 weeks). Patients were followed up every three months until one year, after which they were reviewed every six months.

Pre-operative and follow-up functional outcomes and mobility were objectively recorded using the 30-point system developed by the Musculoskeletal Tumor Society (MSTS) [13]. Radiographs were evaluated for graft consolidation according to the International Society of Limb Salvage [14]. Though this system is used to evaluate allograft, we used it to evaluate our strut autograft. This system uses seven criteria: 1) fusion, 2) resorption, 3) fracture, 4) graft shortening, 5) fixation, 6) joint narrowing, and 7) change in the subchondral bone. Joint narrowing and change in subchondral bone are related to osteoarticular allograft, so these two were not included in the evaluation. The outcome was classified into excellent, good, fair, and poor according to the system.

## Results

The patient population’s mean (SD) age was 24.04 (4.73) years with an age range of 18–34 years. Out of these 25 patients, there were 14 females and 11 males. The mean (SD) of follow-up period (in months) is 30.24(4.38) months with a range of 24–40 months. Out of the 14 patients who had involvement of distal femur, there were eight females and six males. The 11 patients with involvement of proximal tibia included six females and five males. Summary of demographic data and results of our patients are shown in Table 1.

**Table 1:** Summary of demographic data and results from the study.

Sr no.	Age in years	Gender	Anatomical location	Follow up in months	MSTS scores	Range of motion	Campanacci grading
1	24	Female	Distal femur	30	28	0–120	2
2	19	Female	Proximal Tibia	26	30	0–120	2
3	27	Male	Distal Femur	24	22	0–90	2
4	32	Female	Distal Femur	36	24	0–100	2
5	18	Female	Proximal Tibia	30	27	0–120	2
6	25	Male	Distal Femur	24	25	0–90	3
7	21	Female	Distal Femur	28	26	0–110	3
8	29	Male	Proximal Tibia	25	28	0–120	2
9	18	Female	Proximal Tibia	31	26	0–120	2
10	22	Female	Distal Femur	28	15	20–60	3
11	26	Male	Proximal Tibia	30	26	0–110	3
12	24	Female	Distal femur	36	24	0–110	3
13	21	Male	Distal femur	26	28	0–120	2
14	23	Male	Proximal Tibia	28	20	20–90	2
15	27	Female	Distal femur	30	25	0–120	3
16	28	Female	Proximal Tibia	28	27	0–120	2
17	19	Male	Distal femur	34	25	10–100	3
18	23	Female	Proximal Tibia	32	28	0–120	2
19	18	Female	Distal femur	36	27	0–120	2
20	19	Male	Proximal Tibia	26	27	0–120	2
21	22	Female	Proximal Tibia	40	25	0–110	2
22	29	Male	Proximal Tibia	32	25	0–110	3
23	32	Male	Distal femur	38	20	10–90	3
24	34	Male	Distal femur	28	24	0–100	2
25	21	Female	Distal femur	30	28	0–110	2

### Radiological evaluation

There were 16 cases of grade 2 lesions (nine involving proximal tibia and seven involving distal femur) and nine cases of grade 3 lesions (two cases of the proximal tibia and seven cases of the distal femur). Radiological consolidation of the graft was recorded in 24 out of 25 patients. Evaluation of radiographs according to the International Society of Limb Salvage system showed excellent results in 16 patients, a good result in eight, and poor results in one patient. A comparison of patients with involvement of distal femur and proximal tibia is summarized in Table 2.

**Table 2:** Comparison between results of distal femur and proximal tibia GCTs.

	Distal femur GCTs	Proximal tibia GCTs
1. Number of patients	14	11
2. Mean age (SD)	24.78 (5.04)	23.09 (4.34)
3. Gender distribution	6 males, 8 females	5 males, 6 females
4. Campanacci grading	Grade 2 – 7 patients	Grade 2 – 9 patients
	Grade 3 – 7 patients	Grade 3 – 2 patients
5. Mean Follow up period (SD)	30.57 (4.66) months	29.81 (4.16) months
6. MSTS scores	24.35 (3.54)	26.27 (2.53)

### Knee range of motion and Musculoskeletal Tumor Society (MSTS) score

Eighteen patients had complete knee extension and flexion more than 110 degrees, and four patients had complete knee extension with restriction of flexion up to 90 degrees. Three patients had extensor lag and could not achieve full extension. Based on the results of different parameters in the MSTS score for the lower limb, the mean (SD) of MSTS score was 25.2 (3.22) with a range of 15–30. The mean MSTS score for grade 3 tumors was 23.4 compared to 26.18 in cases of grade 2 tumors.

Ten out of eleven patients with involvement of proximal tibia had MSTS score of 25 and above with an average score of 26.27. In contrast, nine out of 14 patients with involvement of distal femur had MSTS scores of less than 25, with an average score of 24.35.

### Complications

**Figure 3:**
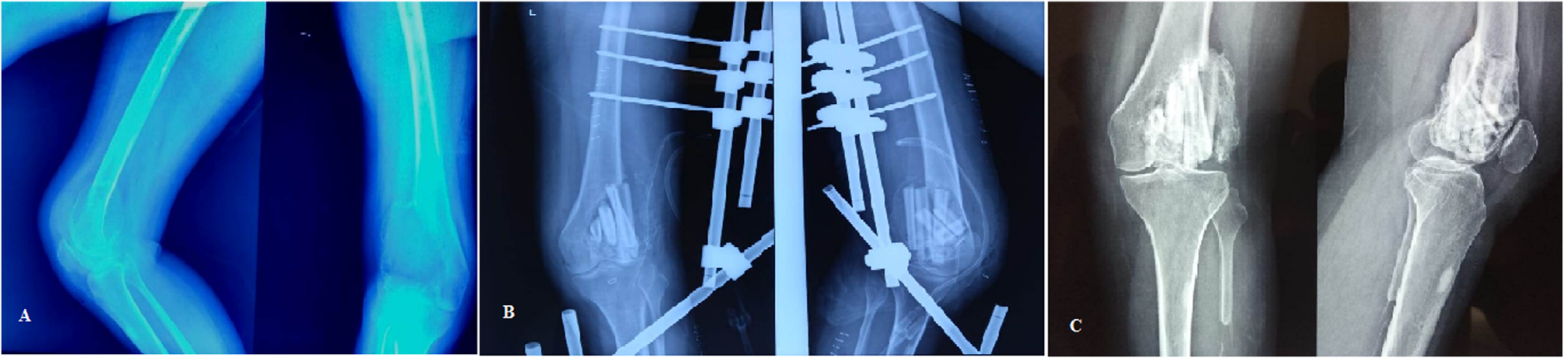
(A) Pre-operative radiographs of a Campanacci grade 3 GCT involving the lateral femoral condyle; (B) Postoperative radiograph; (C) Follow-up radiograph showing good graft consolidation, especially along the posterior cortex. However, one of the fibula struts has breached the articular cartilage and is a cause of persistent knee pain and restricted range of motion.

Three patients had an MSTS score under 20. Two of them had involvement of distal femur, and one of them involved proximal tibia. All three patients had an extension lag with a restricted range of motion (Table 1). One patient of tumor involving the distal femur (Campanacci grade 3) had developed graft subsidence, with fibula penetrating the joint in an antero-posterior radiograph, although it showed good graft consolidation along the posterior and medial cortex (Figure 3). She had persistent pain, stiff knee, and extension lag with a range of motion restricted (20–60 degrees) and MSTS score of 15. However, frank arthritis did not yet develop but was counseled for the need for replacement surgery in the future. Apart from this patient, two other patients had stiff knees at follow-up, as mentioned earlier. However, none of them had any radiographic signs of graft subsidence or arthritis. Five patients had complained of pain around the site where the iliac crest graft was harvested. Two patients had persistent pain over the leg around the fibula graft site. However, the pain was mild and did not hamper their day-to-day activities. None of the patients had any major complications associated with fibula graft harvestings like foot drop, toe extension weakness, or ankle instability. There were no cases of surgical site infection and other wound complications. Five patients had developed a superficial infection around the pin tract that was managed with curettage, pin site dressings, and local antibiotics. No other complications were encountered with the use of external fixators.

## Discussion

Giant cell tumors of the bone are aggressive and potentially malignant primary bone tumors that often occur at the end of the long bone in adults aged 20–40 years old. Surgical Treatment of GCT around the knee focuses on adequate tumor removal and preservation of normal tissue and function. Curettage has a higher recurrence rate [15], but it preserves joint function. Newer chemotherapy agents like denosumab have become a promising option for reducing the tumor bulk and consolidating the tumor before surgery [16, 17]. Its role as an adjuvant when given postoperatively to reduce the chances of recurrence is still controversial. We did not use denosumab in any of our cases because when we operated these cases, it was not in supply at our center.

The study has some limitations, including the retrospective nature of the study, a small number of patients, a modest follow-up period, and excluding the patients of recurrence.

Recurrence rates of procedures involving wide resection and prosthesis are significantly lower compared to joint preservation surgeries. This has been the focus of most of the literature regarding joint preservation surgeries. To avoid this bias, we excluded the patients with tumor recurrence and focused on joint preservation’s clinical and functional outcomes in cases without recurrences.

Based on the data of our study, we observed that 22 patients out of 25 had good functional results with complete knee extension and a satisfactory knee range of motion with MSTS scores averaging 23.4 even in grade 3 cases where there was excessive cortical destruction with thinned out the articular surface. Only one patient in the study developed graft subsidence with fibula graft penetration into the joint. The rest of the complications were mainly related to donor site morbidity and pin tract infection associated with external fixators. Some surgeons have even used casts in place of external fixators and gave similar results [18]. Though not statistically significant, there was a trend of better functional outcomes in cases of GCT involving proximal tibia compared to those involving distal femur. The mean MSTS score may not be very different because of the difference in several patients and the wide variation in scores, the cases involving distal femur had a lesser range of motion and more incidence of knee stiffness. Similarly, the one case that had graft subsidence also had involvement of the distal femur. We opine that the surgical approach which requires parapatellar arthrotomy for distal femur lesions may be the reason for higher incidences of knee stiffness. This aspect warrants further investigations using more patients as it will directly impact the functional outcomes.

After curettage and local ablative therapies, the next main component of joint preservation surgery is to fill the defect. Various methods have been used, including only autograft, only bone cement [9, 10], cement-cancellous bone combination (sandwich technique) [19, 20], allograft and demineralized bone matrix, and even bone substitutes. One apprehension among surgeons regarding the use of autograft alone is the need for abundant bone graft to fill the defect, leading to increased donor site morbidity. We did not fill the entire defect in any of our cases and used only 3–4 fibula struts and some cancellous bone from the iliac crest just below the cartilage layer. All the patients had very good graft consolidation and uptake. In the case of graft subsidence, only one of the fibula struts had penetrated the joint while the rest were nicely incorporated with the posterior and medial cortex (Figure 3). In a study by Prosser et al. [21], they observed that the defects gradually filled with new bone in the majority of the cases, even when they were left empty after curettage. We used a combination of iliac crest and fibula strut graft, as we believe that using only strut graft, especially in the thin subchondral bone, may increase the chances of subsidence and penetration into the joint. At our center, we prefer to put in morselised iliac crest graft in the subchondral bone and create a bed over which strut grafting is done. Based on our experience and the results of our study, we opine that the biological potential of the cancellous bone in metaphysis is underestimated. We now realize that allografts are better options in these cases where a large amount of graft was required without donor site complications. However, we did not have a functioning bone bank at our center when these cases were operated. Some authors have suggested that the use of cancellous bone increases the chances of recurrence [11, 12, 22]. Many surgeons have used bone cement alone or in combination with autograft to fill the defect with good results and lower recurrence rates, and it is difficult to diagnose recurrence by using bone graft alone compared to cement. However, cementing has its own complications like thermal damage to articular cartilage and joint degeneration, the formation of the radiolucent zone around cement, and the loosening of cement leading to micro-motion causing fracture, and local allergic reactions persistent discharge from the wound. Based on our experience, we have stopped using cement and prefer bone grafts alone with augmented curettage, giving us satisfactory results with recurrence rates of about 15%. And in those cases that require revision surgeries, the procedure is easier if there is no cement. Also, we have not seen any patients with fractures due to recurrence when using bone grafts alone but have seen and managed such cases with cement [23–25].

Pathologic fracture is a relatively infrequent complication of giant cell tumor of bone with a variable incidence of around 10% at the time of presentation. It was commonly believed that pathologic fracture was associated with a higher recurrence risk due to the expected contamination of surrounding tissues [26]. However, more recent studies could not confirm pathologic fractures as a risk factor for local recurrence [23]. Furthermore, articular resection may result in significant morbidity and functional impairment. Based on the literature, curettage with adjuvants is a reasonable option for giant cell tumors of bone with pathologic fractures. Resection should be considered with soft tissue extension, fracture through a local recurrence, or when structural integrity cannot be regained after reconstruction [27]. However, given the need for extensive procedures and more rigid internal fixation options required for stabilizing fractures and achieving union, we excluded these cases from our study as its results cannot be directly comparable to those without fracture.

Recently there is an unfortunate trend of increased use of this endoprosthesis in young patients even with benign pathologies (though locally aggressive), neglecting the long-term problems it may create for good short-term outcomes. However, there are a lot of flaws in this approach. Firstly, these tumor prostheses should be used as one of the last tools in the armamentarium and not when joint salvage can be attempted. These prostheses act as a cushion to fall back when joint salvage surgeries fail or there is a recurrence needing more extensive procedures. Using them as the first line leaves us with very few options for the future. Any prosthesis is associated with infection, peri-prosthetic fractures, and the need for revision surgery due to implant loosening in the future. Given the young age of presentation and expected survival of more than 40–50 years with the current trend of life expectancy even in developing countries, we can imagine the number of revision surgeries the patient may require and the risk of infection and peri-prosthetic fractures during his lifetime. Also, the outcome and survivorship of this endoprosthesis are quite dismal, more so when complicated by infections and peri-prosthetic fractures. Secondly, the economics of using these endoprosthesis and frequent revision surgeries and management of complications may take a huge toll on the patient’s financial stability and affect him and his family. This problem is more pronounced in developing countries where healthcare expenditure is mostly borne by the individual rather than insurance based. We have seen in our practice a few patients requesting amputation rather than undergoing multistage procedures for infection and revision. In this study, we observed that joint preservation surgery, using just autograft and external fixators, can give excellent results and obviate the need for complex replacement procedures. Though a few patients may develop some knee range of motion restriction, especially the terminal flexion, that seems to be a fair trade-off compared to endoprosthesis complications.

The findings from these studies reiterate that biological processes should be trusted even when radiographs and tumor stagings do not paint a rosy picture, especially in non-malignant tumors of the young.

## Conclusion

Joint preservation surgery, when done in line with the basic principles of tumor surgery, gives good radiographic and functional outcomes even in grade 2 and 3 giant cell tumors of bone around the knee and should be attempted before replacement surgeries. Filling the entire defect with bone graft is not necessary for graft consolidation.

## Conflict of interest

The authors declare that they have no conflict of interest.

## Funding

The authors received no funding/grant from any external agencies.

## Ethical approval

The study was conducted with the approval of the Institutional review board. Ethical approval was not required, considering the retrospective nature of the study.

## Data availability

The authors confirm that the data supporting the findings of this study are available within the article or its supplementary materials.

## Consent to participate

Written and informed consent was taken from the patient and their family members for using his/her individual and clinical data for publication and research purposes.

## Author contributions

*Saurabh Singh*: Supervision; *Alok Rai*: Conceptualisation, Writing – Review and editing; *R Dinesh Iyer*: Methodology, Writing – Original draft; *Rishabh Surana*: Data curation; *Divyansh Sharma*: Writing – Review and editing.
